# Relationships between muscle strength and multi-channel surface EMG parameters in eighty-eight elderly

**DOI:** 10.1186/s11556-018-0192-z

**Published:** 2018-04-11

**Authors:** Kohei Watanabe, Motoki Kouzaki, Madoka Ogawa, Hiroshi Akima, Toshio Moritani

**Affiliations:** 1 Laboratory of Neuromuscular Biomechanics, School of International Liberal Studies, Chukyo University, Yagotohonmachi, Showa-ku, Nagoya, 466-8666 Japan; 2 Laboratory of Neurophysiology, Graduate School of Human and Environmental Studies, Kyoto University, Kyoto, Japan; 3 Graduate School of Education and Human Development, Nagoya University, Nagoya, Japan; 4 Japan Society for the Promotion of Science, Tokyo, Japan; 5 Research Center of Health, Physical Fitness & Sports, Nagoya University, Japan raduate School of Education and Human Development, Nagoya University, Nagoya, Japan; 6 Faculty of Sociology, Kyoto Sangyo University, Kyoto, Japan; 7 School of Health and Sports Sciences, Chukyo University, Nagoya, Japan

**Keywords:** Aging, High-density surface electromyography, Quadriceps femoris muscles

## Abstract

**Background:**

Since age-related muscle strength loss cannot be explained solely by muscle atrophy, other determinants would also contribute to muscle strength in elderly. The present study aimed to clarify contribution of neuromuscular activation pattern to muscle strength in elderly group. From 88 elderlies (age: 61~ 83 years), multi-channel surface electromyography (EMG) of the vastus lateralis muscle was recorded with two-dimensional 64 electrodes during isometric submaximal ramp-up knee extension to assess neuromuscular activation pattern. Correlation analysis and stepwise regression analysis were performed between muscle strength and the parameters for signal amplitude and spatial distribution pattern, i.e., root mean square (RMS), correlation coefficient, and modified entropy of multi-channel surface EMG.

**Results:**

There was a significant correlation between muscle strength and RMS (*r* = 0.361, *p* = 0.001) in the elderly. Muscle thickness (*r* = 0.519, *p* < 0.001), RMS (*r* = 0.288, p = 0.001), and normalized RMS (*r* = 0.177, *p* = 0.047) were selected as major determinants of muscle strength in stepwise regression analysis (*r* = 0.664 in the selected model).

**Conclusion:**

These results suggest that inter-individual difference in muscle strength in elderly can be partly explained by surface EMG amplitude. We concluded that neuromuscular activation pattern is also major determinants of muscle strength on elderly in addition to indicator of muscle volume.

## Background

Age-related decline in muscle strength is generally greater than decline in muscle mass with aging [29]. This means that while morphological factors are major predictors of muscle strength in human including elderly [31, 42, 43], other causes also contribute to age-related decline in muscle strength. In many possible contributors, neuromuscular function would be one of the important factors since voluntary muscle contraction is regulated by neuromuscular activation system. Similarly to age-related morphological changes, modifications with aging in neuromuscular activation pattern have been reported in previous studies, i.e., decrease in motor unit firing rate [10, 20, 34, 38]. However, little is known about relationship between decline in muscle strength and modification in neuromuscular activation pattern for elderly whereas many studies have demonstrated that age-related decline in muscle strength is strongly associated with morphological changes in aged muscle [31, 42, 43]. This suggests that contribution of alteration in neuromuscular activation pattern to decline in muscle strength with aging is not well understood. It can be partly explained by methodological limitation to measure and quantify neuromuscular function.

Surface electromyography (EMG) has been widely used for measuring and/or quantification of neuromuscular function since this method can non-invasively detect motor unit action potentials which is a trigger of important physiological process in muscle contraction [2, 7, 15]. On the other hand, this methodology cannot investigate detailed motor unit activation properties, such as firing rate or recruitment threshold of individual motor units, since the detected signals are the result of summation of action potentials from multiple motor units under the electrodes [13, 15]. Intramuscular EMG is a limited tool for direct detecting of individual motor unit action potentials [9, 25], but is not useful for various types of subjects such as elderly because of invasiveness. Instead of these methodologies, some studies including our studies recently apply multi-channel surface EMG technique to estimate motor unit recruitment pattern from spatial distribution pattern of action potential within a muscle [11, 14, 17, 18, 39–41]. From a spatial inhomogeneity in the location of different types of muscle fibers and a clustering of muscle fibers innervated by one motor unit in limited territory [3, 4, 22, 23, 35], alteration in spatial distribution pattern of surface EMG within a muscle would be available to estimate motor unit recruitment pattern. Holtermann et al. [17] and Farina et al. [14] demonstrated that changes in spatial distribution pattern of surface EMG within a muscle with an increase of contraction level and fatigue for estimating motor unit recruitment patterns [14, 17]. Our previous study demonstrated that lesser change in spatial distribution pattern of surface EMG within vastus lateralis (VL) muscle with an increase in exerted force for elderly comparing with young [39]. This phenomenon is seemed to be fitted with age-related morphological changes in skeletal muscle, such as decrease in number of motor unit, increase in innervation ratio, or remodeling of motor units from fast to slow types [8, 21–23, 33, 35]. However, relationship between muscle strength and/or age-related muscle weakness and neuromuscular functions estimated from spatial distribution pattern of surface EMG are not investigated because of small sample size and limited range of muscle strength for elderly subjects.

We aimed to clarify contribution of neuromuscular activation pattern to muscle strength in elderly group by using multi-channel surface EMG variables. In the present study, spatial distribution pattern of surface EMG within the VL muscle were used to determine the degree of association with muscle strength and were compared among subject group of elderly with different muscle strength and young subjects. Power et al. [33] showed that elderly with lifelong high-intensity physical activity could mitigate age-related loss of motor units. Other studies demonstrated changes in motor unit activation properties such as EMG amplitude or firing rate following resistance training even in aged muscle [19, 30]. These studies suggest motor unit remodeling and/or modification of motor unit activation properties with aging could be varied among individuals and depend on amount of lifelong physical activity. From age-related alteration in spatial distribution pattern of surface EMG could be partly explained by motor unit remodeling and modification of motor unit activation properties with aging, we hypothesized that motor unit recruitment pattern which is estimated from multi-channel surface EMG variables could shed some light upon inter-individual difference in muscle strength in elderly.

## Methods

### Subjects

A total of 111 subjects including 88 elderlies (age: 61~ 83 years) and 23 young (age: 19~ 26 years) participated in this study. The subjects living in a nursing home were included in the elderly group. The young subjects were male university students and not participated in any competitive sports events. Twenty-seven females were included in elderly group.

The subjects gave written informed consent for the study after receiving a detailed explanation of the purposes, potential benefits, and risks associated with participation. All procedures used in this study were approved by the Committee for Human Experimentation at the Graduate School of Human and Environmental Studies, Kyoto University and the Research Ethics Committee of Chukyo University, and were in accordance with the Declaration of Helsinki.

### Experimental design

To familiarize themselves with the motor tasks used in the present study, all subjects came to the laboratory > 1 week before the experimental day. The subjects performed maximal voluntary contraction (MVC) and submaximal incremental ramp contraction during unilateral isometric knee extension. External force at the distal portion of the shank that fixed in a custom-made dynamometer (Takei Scientific Instruments Co., Ltd., Niigata, Japan) with a force transducer (LU-100KSE; Kyowa Electronic Instruments, Tokyo, Japan) was measured during isometric knee extension. During knee extension, both hip and knee joint angles were flexed at 90° (180° corresponds to full extension). Since European consensus on definition and diagnosis used this knee joint angle [6], the present study selected this knee joint angle.

The MVC trial consists of a gradual increase in knee extension force from baseline to maximum in 2–3 s and a plateau phase at maximal contraction for 2–3 s. The timing of the task was based on a verbal count given at 1-s intervals with vigorous encouragement from the investigators. After three submaximal trials at approximately 50, 70, and 90% of MVC as warming up, the subjects performed two MVC trials with ≥ 2 min rest in between. MVC force was mean value of 1 s, when the highest force was produced, during a plateau phase. MVC torque was calculated as the product of MVC force and the distance between the estimated knee joint center and center of force transducer at the distal portion of the shank. To normalize difference in body type among the subjects, MVC torque relative to body mass (MVC/BM) was also calculated and used for further analysis.

**Fig. 1:**
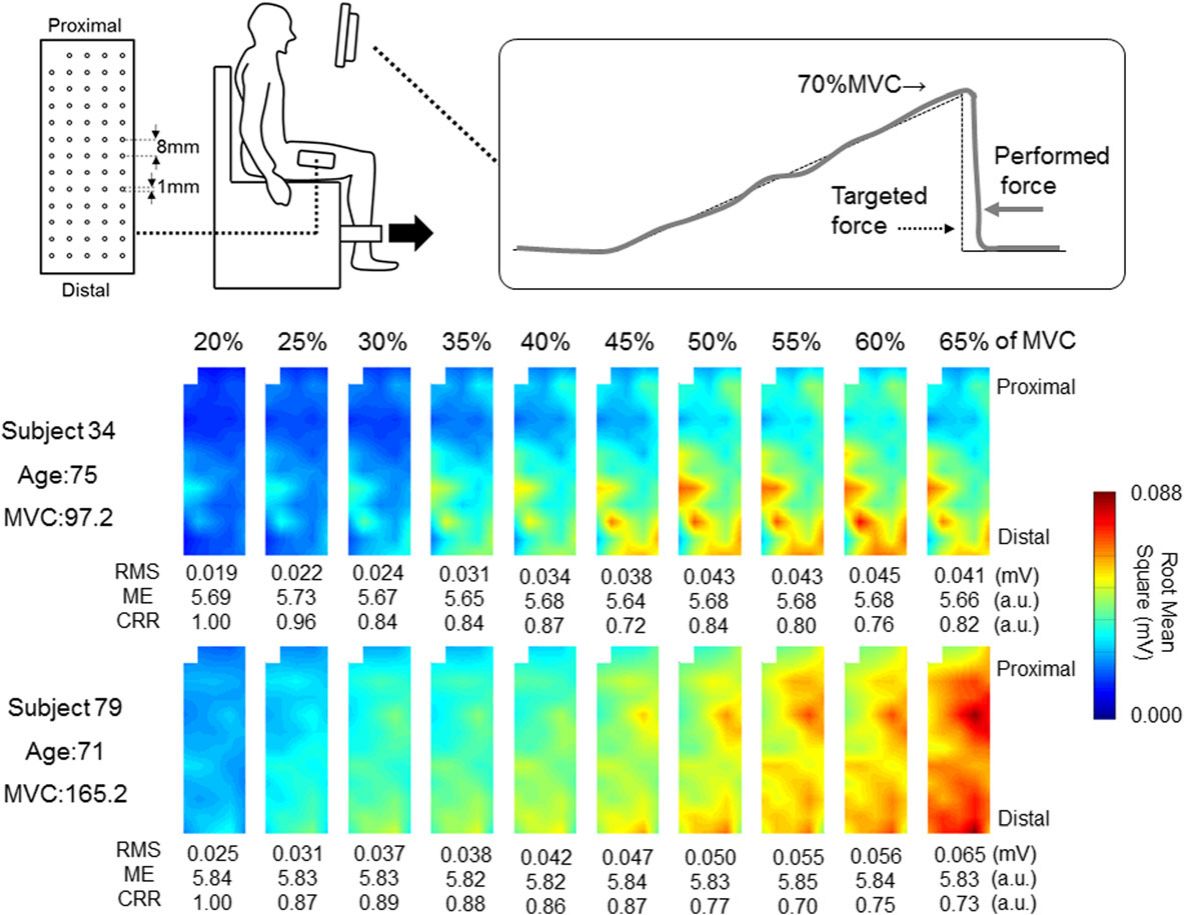
Electrode, experimental setup, and motor task used in the present study and representative spatial distribution of root mean square of multi-channel surface electromyography in vastus lateralis muscle shown as color map for two elderlies with different muscle strength. MVC: maximal voluntary contraction

After MVC trials, the subjects performed submaximal ramp contractions from 0 to 70% MVC in 35 s (rate of force increase: 5% MVC/s) (Fig. 1). Target and performed forces were shown to the subjects on a monitor. Two submaximal contractions were performed with ≥ 2 min rest in between. Out of two trials, the one trial with the smaller error between the targeted and performed forces was selected with visual inspection and used for analysis.

### Multi-channel surface EMG

Surface EMG signal was recorded from the VL muscle with a semi-disposable adhesive grid of 64 electrodes (ELSCH064R3S, OT Bioelectronica, Torino, Italy). The two-dimensional electrode grid is comprised of 13 rows and 5 columns of electrodes with 1 missing electrode in the upper left corner. Each electrode is 1-mm diameter and inter electrode distance was 8-mm inter-electrode distance in both directions (Fig. 1). Before attaching the electrode, the skin was cleaned with alcohol. The line between the head of the great trochanter and inferior lateral edge of the patella which was defined and used as reference line for positioning the electrode grid. The center of electrode grid was positioned at middle point of the reference line and columns of electrodes aligned with the reference line. The position of the missing electrode was located proximally. To assure proper electrode contact with the skin, conductive gel was inserted into the cavities of the grid electrode. The grid electrode was connected to the amplifier through 4 connectors which were fixed to the skin by elastic tape. A reference electrode (C-150, Nihon Kohden, Tokyo, Japan) was placed at the iliac crest.

Fifty-nine bipolar surface EMG signals along the columns were made from 64 electrodes. Root mean square (RMS) values were calculated from rom bipolar signal sampled over 1 s at 5% increment from 20 to 65% of MVC ramp contraction. Sampled signals were overlapped by 0.5 s between neighboring contraction levels, since performed ramp rate was 10% of the MVC force / 1 s.

We used correlation coefficients and modified entropy to compare spatial distribution pattern and to quantify a heterogeneity for RMS values of multi-channel surface EMG within a grid, respectively. Correlation coefficients were calculated from the 59 pairs of RMS values at the same regions between 20% of MVC and those of all other torque levels to compare the spatial EMG potential distribution pattern as used in our previous studies [40]. Decrease of correlation coefficient indicates change in spatial EMG potential distribution pattern. Modified entropy was calculated as done by Farina in a previous work [14] and our previous studies [39–41], modified entropy was defined as entropy of the signal power, that is.

$$ E=-{\sum}_{i=1}^{59}p{(i)}^2{\log}_2p{(i)}^2 $$

where *p(i)* is the square of the RMS value of channel *i* divided by the sum of the squares of all the 59 RMS values, at the given force level. Therefore *p(i)*^*2*^ represents the normalized power of each channel. It is E = 0 when all the *p(i)* are zero except one and is maximal and equal to *log*_*2*_*59 = 5.884* when the *p(i)* values are identical and equal to 1/59 (all channels have the same energy). Decrease in modified entropy mean that increase of heterogeneity in spatial EMG potential distribution within an electrode grid.

At the center of electrode location, longitudinal ultrasonographic images (SSD-900, ALOKA, Tokyo, Japan, or Fazone CB, Fuji-film, Tokyo, Japan) were taken to measure the thickness of the subcutaneous tissue and VL muscle before attaching the electrode grid. On NIH ImageJ software, the vertical line of the image was defined on the center of horizontal axis for measurements. Distance between the intersections of that vertical line and deeper and superficial aponeurosis of the VL muscle were measured as muscle thickness of VL. Thickness of subcutaneous tissues was distance between the intersections of that vertical line and superficial aponeurosis of the VL muscle and skin surface.

### Statistics

All data are provided as the mean and standard deviation. To test relationship between muscle strength and neuromuscular function, spearman’s rank correlation coefficient was calculated for MVC with multi-channel surface EMG variables and others for the elderly and young groups, respectively. We also performed a stepwise regression analysis for MVC in the elderly group. Nine independent variables, i.e., age, muscle thickness of VL, thickness of subcutaneous tissues, and six surface EMG variables, were entered the stepwise regression if they represented a significant contribution to the explained variance corresponding to an alpha level of *p* < 0.05. Six EMG variables were modified entropy, correlation coefficient value, mean RMS across all 59 channels, standard deviation of RMS across all 59 channels, mean RMS across all 59 channels normalized by those at 20% of MVC (normalized RMS), and standard deviation of mean RMS across all 59 channels normalized by those at 20% of MVC (normalized standard deviation) and these variables calculated at 65% of MVC were used for correlation analysis.

To compare neuromuscular function among the elderly with different muscle strength, 88 elderly subjects were divided into three groups based on MVC using cumulative frequency distribution, i.e., (Weak) < 33.3%, (Mid) 33.4~ 66.6%, and (Strong) > 66.7%, and the weak and strong groups were used for further analysis. Age, height, body mass, MVC, MVC/BM, muscle thickness of VL, and thickness of cutaneous tissues, and modified entropy, correlation coefficient value, and mean RMS across all 59 channels at from 20 to 65% of MVC were compared between the weak and strong strength groups by using Mann-Whitney test. EMG variables for the elderly groups with weak and strong strength were compared with young subjects by using Mann-Whitney test.

We also compared modified entropy, correlation coefficient value, and mean RMS across all 59 channels at from 20 to 65% of MVC between the elderly and young groups which are matched for MVC. MVC/BM, body mass, muscle thickness of VL, and thickness of cutaneous tissues were also matched between these groups.

The level of significance was set at *p* < 0.05. Statistical analysis was performed using SPSS (version 15.0, SPSS, Tokyo, Japan) and MATLAB (R2009b, MathWorks GK, Tokyo, Japan).

## Results

Representative multi-channel surface EMG from 20% to 65% of MVC for two subjects with high and low muscle strength were shown as two-dimensional map of color scaled RMS (Fig. 1). With increase in exerted force, RMS of each channel non-uniformly increased within an electrode grid in both subjects and higher RMS were seen in subject 79 than subject 34. While increase in RMS is represented at limited area (distal portions) in subject 34, increase in RMS were demonstrated in larger area and spatial distribution pattern of RMS markedly changed in subject 79. This was reflected as lower correlation coefficient value at 50~ 65% of MVC in subject 79. Also, area of similar RMS seemed to be larger in subject 79 at all contraction levels comparing with subject 34, reflecting higher modified entropy at each force level in subject 79.

### Correlation analysis

**Fig. 2:**
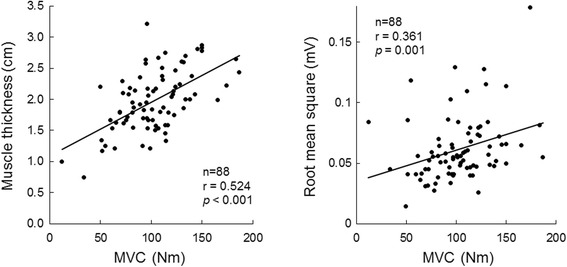
The relationships with maximal voluntary contraction of knee extension joint torque for muscle thickness (left panel) and root mean square of surface electromyography (right panel) in the vastus lateralis muscle for 88 elderly subjects. MVC: maximal voluntary contraction

There were significant correlations with MVC in muscle thickness of VL (*p* < 0.001) (Fig. 2 left), RMS (*p* = 0.001) (Fig. 2 right), and normalized RMS (*p* = 0.003) in the elderly group (Table 1).

**Table 1:** Correlation coefficient analysis in elderly (*n* = 88)

		R	P	
MVC	Age	− 0.111	0.324	
	MT	0.524	< 0.001	*
	ST	−0.150	0.182	
	ME	0.056	0.622	
	RMS	0.361	0.001	*
	NRMS	0.327	0.003	*
	CRR	−0.180	0.108	

MVC, maximal voluntary isometric knee extension joint torque; MT, Muscle thickness of vastus lateralis; ST, subcutaneous tissue thickness; ME, modified entropy of surface electromyography at 65% of the maximal voluntary contraction; RMS, root mean square value of surface electromyography for all channels in electrode grid at 65% of the maximal voluntary contraction; NRMS, root mean square of surface electromyography for all channels in electrode grid at 65% of the maximal voluntary contraction normalized by that at 20% of the maximal voluntary contraction; CRR, correlation coefficient value in root mean square of surface electromyography between 20 and 65% of the maximal voluntary contraction

* indicates significant correlation

Significant correlations with MVC were found in modified entropy (*p* = 0.004) in the young group (Table 2).

**Table 2:** Correlation coefficient analysis in young (*n* = 23)

		R	P	
MVC	Age	0.357	0.095	
	MT	0.326	0.129	
	ST	0.291	0.178	
	ME	−0.583	0.004	*
	RMS	0.406	0.054	
	NRMS	−0.086	0.697	
	CRR	0.201	0.359	

MVC, maximal voluntary isometric knee extension joint torque; MT, Muscle thickness of vastus lateralis; ST, subcutaneous tissue thickness; ME, modified entropy of surface electromyography at 65% of the maximal voluntary contraction; RMS, root mean square value of surface electromyography for all channels in electrode grid at 65% of the maximal voluntary contraction; NRMS, root mean square of surface electromyography for all channels in electrode grid at 65% of the maximal voluntary contraction normalized by that at 20% of the maximal voluntary contraction; CRR, correlation coefficient value in root mean square of surface electromyography between 20 and 65% of the maximal voluntary contraction

* indicates significant correlation

In stepwise regression analysis for the elderly group, muscle thickness of VL, RMS, and normalized RMS were selected from ten independent variables influencing MVC (Table 3). The final regression equation was:

**Table 3:** Stepwise regression analysis for elderly

MVC = 34.619*MT + 349.554*RMS + 8.921*NRMS *R* = 0.664, R^2^ = 0.441, Adjusted R^2^ = 0.419			
Dependent variables	Independent variables	Standardized regression coefficient	P
MVC	MT	0.519	< 0.001
	RMS	0.288	0.001
	NRMS	0.177	0.047

MVC, maximal voluntary isometric knee extension joint torque; MT, Muscle thickness of vastus lateralis; RMS, root mean square of surface electromyography; NRMS, root mean square of surface electromyography for all channels in electrode grid at 65% of the maximal voluntary contraction normalized by that at 20% of the maximal voluntary contraction

MVC = 34.619 x muscle thickness of VL + 349.554 x RMS + 8.921 x normalized RMS.

The correlation coefficient R, R^2^ and adjusted R^2^ for this model was 0.664, 0.441, and 0.419, respectively. Also, there were no multicollinearity (Variance Inflation factor < 10.0, for all) among the selected variables, meaning that the selected variables are independent.

### Comparisons among different muscle strength in elderly

**Fig. 3:**
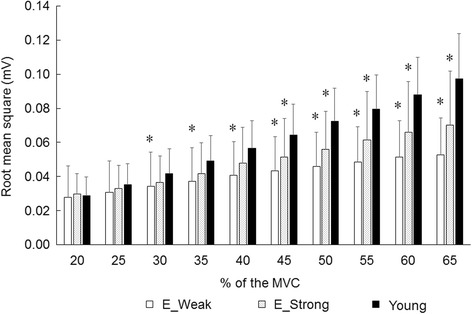
Root mean square of multi-channel surface electromyography in vastus lateralis muscle for the elderly groups with different muscle strength and young. MVC: maximal voluntary contraction, E_Weak: elderly group with weak strength, E_Strong: elderly group with strong strength, *: significant difference with young group

**Fig. 4:**
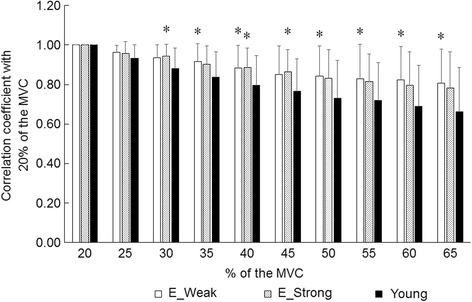
Correlation coefficient with 20% of maximal voluntary contraction of multi-channel surface electromyography in vastus lateralis muscle for the elderly groups with different muscle strength and young. MVC: maximal voluntary contraction, E_Weak: elderly group with weak strength, E_Strong: elderly group with strong strength, *: significant difference with young group

**Fig. 5:**
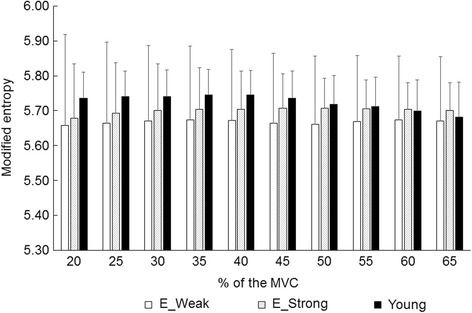
Modified entropy of multi-channel surface electromyography in vastus lateralis muscle for the elderly groups with different muscle strength and young. MVC: maximal voluntary contraction, E_Weak: elderly group with weak strength, E_Strong: elderly group with strong strength, *: significant difference with young group

Elderly with < 91.5 Nm (*n* = 27) and > 113.9 Nm (*n* = 28) of MVC were selected as weak and strong strength groups. There were significant differences in height, body mass, MVC, MVC/BM, and muscle thickness of VL (*p* < 0.05). No significant differences were found in RMS, correlation coefficient value, and modified entropy across all 59 channels at from 20 to 65% of MVC between the elderly groups with weak and strong strength (*p* > 0.05) (Figs. 3, 4 and 5).

### Comparisons between elderly and young groups

In comparisons to young group for multi-channel surface EMG parameters, significant differences in RMS were observed for the weak and strong groups at 45–65% of MVC (*p* < 0.05) (Fig. 3). In correlation coefficient with 20% of MVC, there were significant differences with young group for the weak strength group at 35%, 40%, and 50–65% of MVC and for the strong strength group at 30% and 40% of MVC (*p* < 0.05) (Fig. 4).

Ten elderly men (67.6 ± 3.9 ys) and nine young men (21.1 ± 1.1 ys) were selected as the MVC -matched elderly and young groups. Between the MVC-matched elderly and young groups, significant differences were seen in RMS at 20% and 25% of MVC (p < 0.05). There were no significant differences in modified entropy and correlation coefficient value of surface EMG (*p* > 0.05).

## Discussion

The present study aimed to clarify contribution of neuromuscular activation pattern to muscle strength in elderly group by using multi-channel surface EMG variables. Our main findings in the present study were that 1) maximal muscle strength in elderly was positively correlated with surface EMG amplitude (Table 1), 2) inter-individual difference of muscle strength in elderly can be explained by muscle thickness and surface EMG amplitude (Table 3), 3) there were significant differences in multi-channel surface EMG parameters with young for elderly with weak strength at middle to high force levels and for the elderly with strong strength at middle force levels (Fig. 4), and 4) absolute muscle strength-matched elderly and young groups shows similar multi-channel surface EMG parameters. Taken together, inter-individual difference in muscle strength for elderly is related with amplitude of surface EMG and the elderly with weak and strong strength have different manners in multi-channel surface EMG parameters. These results partly support our hypothesis that motor unit recruitment pattern which is estimated from multi-channel surface EMG variables can explain inter-individual difference in muscle strength in elderly.

### Positive correlation between muscle strength and surface EMG amplitude in the elderly

In the elderly group, muscle thickness, RMS, and normalized RMS were significantly correlated with MVC (*p* < 0.05) (Table 1) and in stepwise regression analysis muscle thickness, RMS, and normalized RMS were selected as determinants of MVC (Table 3). Also, stepwise regression analysis shows high standardized regression coefficient in RMS (0.288, *p* = 0.001) in addition to MT (0.519, *p* < 0.001) (Table 3). Although significant correlation was not observed, tendency to correlate between MVC and RMS was also shown in young (*p* = 0.054) (Table 2). These results suggest that the variables related to neuromuscular function are also major determinants of muscle strength in addition to indicators of muscle volume such as muscle thickness [16, 31, 42, 43].

Since voluntary muscle contraction is regulated by central nervous system, it would be reasonable to conclude that neuromuscular function contributes to individuals’ muscle strength. EMG amplitude variables, i.e., RMS and normalized RMS, mainly reflect number of recruited motor units and its firing rate during voluntary contraction. Decreases in number of motor unit [24, 33] and in motor unit firing rate [34] with ageing are well known. These changes would induce decrease in EMG amplitude in the elderly. Merletti et al. [26] reported a significantly lower averaged rectified value of surface EMG of biceps brachii muscle during submaximal isometric contractions in elderly compared with young [26]. On the other hand, age-related decrease in EMG amplitude may not be uniformly manifested among elderly individuals. Power et al. [33] demonstrated that the estimated number of motor units in tibialis anterior muscle were significantly lower in elderly than young, but not in master runners [33]. In our recent study that used a decomposition of motor units action potential from multi-channel surface EMG, positive correlation between MVC and motor unit firing rate was demonstrated in the VL muscle for elderly [38]. Variation in EMG amplitude among individuals and relationship between EMG amplitude and muscle strength in the elderly may be brought about by these inter-individual differences in age-related alterations for anatomy and/or regulation of motor units [24, 33, 34, 38]. However, we should note that surface EMG is strongly influenced by geometric or non-physiological parameters [12, 27]. When amplitude variables are compared among the individuals, differences in the thickness of the subcutaneous tissue should be considered [28]. To quantify the effect of inter-individual differences in the subcutaneous tissue on relationship between EMG amplitude and muscle strength, we additionally calculated partial correlation coefficient between RMS and MVC adjusted for the thickness of the subcutaneous tissue in the elderly. Adjusted correlation coefficient (*r* = 0.291, *p* = 0.009) was slightly lower than the value before adjustment (*r* = 0.361, *p* = 0.001). While this means that relationship between MVC and RMS was influenced by the thickness of the subcutaneous tissue, significance in correlation coefficient value is unchanged. We thus assumed that the thickness of the subcutaneous tissue was not critical effect on our results.

### Relationship between muscle strength and estimated motor unit recruitment pattern

In the present study, different manners in multi-channel surface EMG parameters were observed between the elerly with weak and strong strength when compared to the young subjects (Fig. 4). Significant differences in correlation coefficient values with the young subjects were noted at high force levels in the elderly with weak strength (< 91.5 Nm) (*p* < 0.05), but not in the elderly with strong strength (> 113.9 Nm) (*p* > 0.05) (Fig. 4). Also, as the results of comparison between the young and elderly under the condition that MVC were matched, no significant difference was observed in correlation coefficient values at all contraction levels (*p* > 0.05). Correlation coefficient value between RMS from same channels between 20% of MVC and other force levels was used to assess changes in spatial distribution of surface EMG within a muscle in the present study. Holtermann et al. [18] showed that alteration pattern of correlation coefficients during ascending and descending phases during ramp contraction was not the same [18]. Since a deviation between recruitment and derecruitment of motor units was reported [18], the correlation coefficient of multi-channel SEMG could be reasonably used to estimate recruitment of global motor units during force production. From the results in the present study, it can be interpreted that changes in spatial distribution patterns of RMS from 20% to 65% of MVC were lesser in the elderly with weak muscle strength and were similar in the elderly with strong muscle strength when compared to the young. Based on our assumption, motor unit recruitment pattern of the young may be different from the elderly, which is corresponds to the conclusion in our previous study [39], but not from the elderly with high muscle strength. The results of some previous studies had pointed out that importance of neural factors in muscle strength for elderly. Previous studies reported that neural activation is more adaptable for the resistance training in elderly than young [5, 19, 30]. The results of the present study could support this notion and suggests that neural factor should be also evaluated for understanding age-related muscle strength loss in addition to indicator of muscle volume.

In the young subjects, significant correlation with MVC was noted only in modified entropy of multi-channel surface EMG (Table 2). Lower modified entropy means greater heterogeneity of spatial distribution of surface EMG amplitude within an electrode grid. Our previous study demonstrated lower modified entropy at 65% of MVC in the young compared with the elderly [39]. From this result, we estimated that spatial inhomogeneity in location of different types of muscle fibers/motor units and/or numbers of newly recruited muscle fibers/motor units are greater in the young. Therefore, the result of the present study suggest that motor unit recruitment pattern strongly relates with the muscle strength in young and the determinants of muscle strength are not same between the young and elderly.

### Limitations

The present study used a specific knee joint angle. Although the knee joint angle of 90° has been widely used in the related research area [6], the highest knee extension torque is observed at more extended knee joint angles (115°-140°) [36, 37]. We also should note that surface EMG and muscle thickness were measured only from the VL muscle in the present study. Knee extension joint torque is produced by four muscle components of quadriceps femoris, i.e., the VL, vastus intermedius, vastus medialis, and rectus femoris muscles. Neuromuscular activation pattern are not uniform among the four muscle components and their contribution to knee extension torque could be influenced by knee joit angle [32, 37]. Therefore, the results of the present study would be specific for one knee joint angle and the VL muscle. Different results may be observed at different joint angles and in other muscles. Also, the present study calculated maximal voluntary joint torque for knee extension from the estimated moment arm length at the resting condition and measured force. Since the soft tissue deforms during loading, the estimated moment arm dinamically change between the resting and contraction conditions [1]. Therefore, our calculated joint torque would not be consistent with the resultant knee joint moment. This is also the major issues of this study.

## Conclusion

The present study aimed to clarify contribution of neuromuscular activation pattern to muscle strength by using multi-channel surface EMG variables in elderly group. We demonstrated that surface EMG amplitude is positively correlated with muscle strength. Also, the elderly with weak and strong strength have different manners in the indicators of spatial distribution pattern of multi-channel surface EMG. Our findings suggest that inter-individual difference in muscle strength in elderly can be partly explained by multi-channel surface EMG variables. The present study concluded that neuromuscular activation pattern is also major determinants of muscle strength on elderly in addition to indicator of muscle volume.

## Abbreviations

EMG: Electromyography
MVC: Maximal voluntary contraction
SEMG: Surface electromyography
VL: Vastus lateralis
